# Magnetic resonance imaging performance for diagnosis of ovarian torsion in pregnant women with stimulated ovaries

**DOI:** 10.1186/s40738-017-0040-2

**Published:** 2017-09-06

**Authors:** Elizabeth Asch, Jesse Wei, Koenraad J. Mortele, Kathryn Humm, Kim Thornton, Deborah Levine

**Affiliations:** 1 Department of Radiology, Division of Ultrasound, Brigham and Women’s Hospital, 75 Francis Street, L1, Boston, MA 02115 USA; 2 Department of Radiology, Beth Israel Deaconess Medical Center, 330 Brookline Ave, Boston, MA USA; 3 George Washington University Hospital, 22nd and I Street, NW, 6th Floor, Washington, DC USA; 4 Department of Obstetrics and Gynecology, Beth Israel Deaconess Medical Center, 330 Brookline Avenue, Boston, MA USA

**Keywords:** Magnetic resonance imaging, Ovarian torsion, Ovarian stimulation

## Abstract

**Background:**

To determine if asymmetric ovarian edema on non-contrast MRI can be used to distinguish torsed from non-torsed stimulated ovaries in pregnant women.

**Methods:**

In this retrospective study, our radiology database was searched for women who were pregnant and who had undergone ovarian stimulation and underwent MRI abdomen/pelvis from 1/2000–12/2012. At our institution, ultrasound is typically performed as a first line study for pregnant women with pelvic pain, with MR for those patients with indeterminate findings. 64 pregnant women (gestational age range 3–37 weeks) were included. MRI indication, prospective interpretation, operative diagnosis, and follow-up were recorded. Two blinded radiologists (with a third radiologist tie-breaker) independently measured and described the ovaries, including the likelihood of torsion. If one or both ovaries/adnexa had an underlying lesion such as a dermoid, cystadenoma, or abscess, the patient was excluded from size and signal intensity comparison (*N* = 14). For the remaining 50 women, comparison was made of the ovaries in women with normal ovaries (*N* = 27), stimulated ovaries without torsion (*N* = 11), non-stimulated ovaries with torsion (*N* = 3), and stimulated ovaries with torsion (*N* = 3). Patients with asymmetric ovarian edema without stimulation or torsion (*N* = 3) and with polycystic ovary syndrome (*N* = 3) were analyzed separately.

**Results:**

Average normal ovarian length was 3.2 cm, compared to 4.5 cm for asymmetric edema and 5.6–8.8 cm for the other four groups. Average difference in greatest right and left ovarian diameter was 19% for normal ovaries compared to 24–37% for the other 5 groups. Asymmetric signal on T2-weighted imaging (T2WI) was seen in 12% (3/27) of normal ovaries compared to 9% (1/11) of stimulated patients without torsion, 33% (1/3) of patients with PCOS and 67% (2/3) of patients with torsion both without and with stimulation. The correct diagnosis of torsion was made prospectively in 5/6 cases but retrospectively in only 3/6 cases. In patients with stimulation, correct diagnosis of torsion was made in 2/3 cases prospectively (both with asymmetric T2 signal) and retrospectively in only 1/3 cases. In 13/64 patients, other acute gynecologic and non-gynecologic findings were diagnosed on MRI.

**Conclusions:**

Enlarged edematous ovary can be seen with ovarian stimulation, ovarian torsion, or both. Although asymmetric ovarian edema occurred more frequently in patients with torsion than without, in pregnant patients with stimulated ovaries referred for MRI (typically after non-diagnostic ultrasound), ovarian torsion could not be confidently diagnosed or excluded retrospectively with non-contrast MRI.

## Background

Women undergoing ovarian stimulation are at increased risk for ovarian torsion [1, 2]. The imaging interpretation of these patients’ findings is complicated since ovarian stimulation itself can result in enlarged painful ovaries [3], as does ovarian torsion. When these patients present with acute unilateral pelvic pain, the recommended first line imaging modality for evaluation for ovarian torsion is ultrasound. Ultrasound findings in ovarian torsion include enlargement of the torsed ovary, edematous appearing stroma, peripheralized follicles, and twisted ovarian pedicle [4, 5]. Color and pulsed Doppler can be helpful when the symptomatic side shows lack of flow; however, due to the dual blood supply of the ovaries as well as variable degrees of twisting, torsion can be present even when the blood flow sonographically appears preserved [6]. Normal blood flow is less likely in patients with ovarian torsion who have undergone ovarian stimulation but does not exclude torsion even in this population [7]. The most consistent ultrasound finding of ovarian torsion in the setting of stimulation is asymmetric enlargement of the torsed ovary [8, 9]. However, underlying bilateral ovarian enlargement in the setting of stimulation may be asymmetric without torsion. Therefore, in a patient with stimulated ovaries, unilateral pelvic pain, an enlarged ovary and the presence of ovarian blood flow, ovarian torsion cannot be excluded with ultrasound.

In the setting of an inconclusive ultrasound, magnetic resonance imaging (MRI) is often utilized to evaluate further for ovarian torsion or other causes of acute lower abdominal/pelvic pain, particularly when the patient is pregnant [10]. MRI characteristics of ovarian torsion are similar to ultrasound findings, including ovarian enlargement, peripheralized follicles, stromal edema, hemorrhage, twisted ovarian pedicle, fallopian tube wall thickening, free fluid in the pelvis, and uterine deviation to the side of torsion [4, 5, 11]. However, some of these findings also can be seen in the setting of ovarian stimulation without torsion. Low apparent diffusion coefficient (ADC) values have a high sensitivity for hemorrhagic infarction [11], but early torsion cannot be excluded in the presence of normal ADC values. Hypoenhancement of the ovary is suggestive of torsion [4], but use of gadolinium in pregnancy is typically avoided because it is a pregnancy class C drug [12] and has not been characterized in stimulated ovaries. No MRI characteristic of the ovary that cannot be seen with ultrasound has been shown to increase sensitivity for ovarian torsion. Therefore, laparoscopy is needed for definitive diagnosis and treatment of ovarian torsion. We hypothesized that asymmetric ovarian edema comparing right and left ovaries on non-contrast MRI may be diagnostic for ovarian torsion in pregnancy, particularly in patients with underlying ovarian enlargement in the setting of stimulation.

MRI characteristics of ovarian torsion in patients with adnexal masses have previously been reported [13]. However, the ovaries may have a different appearance and location in pregnant compared to non-pregnant women. In addition, MRI characteristics of ovarian torsion in pregnant patients with stimulated ovaries have not, to our knowledge, been defined. Thus, the purpose of our study was to determine if asymmetric ovarian edema on non-contrast MRI can be used to distinguish torsed from non-torsed stimulated ovaries in pregnant women.

## Methods

This retrospective study was conducted with approval from our institution’s Institutional Review Board and was HIPAA compliant with waiver of informed consent.

Our institution is an urban academic medical center with a busy obstetric and gynecologic service, which serves a large population of women undergoing assisted reproduction, including ovarian stimulation. Gynecologic ultrasound and MRI are both available 24 h a day, 7 days a week. At our institution, most patients undergoing MRI in pregnancy undergo ultrasound first for evaluation of pelvic pain and only pursue MRI if the ultrasound is non-diagnostic.

### Subjects

All MRI abdomen and pelvis examinations performed at our institution between January 2000 and December 2012 were searched in our imaging archive database for the terms “torsion, IVF, stim*, vitro, torted, OHSS, torsed.” Of these cases, all reports containing the terms “ovar*, adnexa” were searched. This yielded 266 MRI examinations in 250 women.

64 pregnant patients (including 2 women who had undergone embryo transfer the day of and the day prior to MRI) underwent 65 MRI examinations during the study period. For the patient who underwent two MRI examinations during the study period, the earlier examination was included in data analysis. One patient whose ovaries were not visualized on MRI was excluded. Because non-pregnant patients can have a contrast enhanced examination and because pregnancy can change the location of the ovaries, which can potentially lead to differences in venous drainage and subsequent differences in ovarian stromal appearance, we used as our comparison population pregnant patients without ovarian stimulation. Patients with diagnoses that could complicate assessment of ovary size or appearance (*N* = 14) were excluded from side to side comparisons. The final study population for side to side comparison included 50 patients: normal ovaries (*N* = 27), asymmetric ovarian edema without torsion or stimulation (*N* = 3), stimulated ovaries without torsion (*N* = 11), torsion without stimulation (*N* = 3), torsion with stimulation (*N* = 3), and polycystic ovary syndrome (PCOS, *N* = 3).

The imaging protocol was tailored when needed for each patient. In general, we followed a previously published protocol for imaging pregnant patients with pelvic pain [14]. In brief, MR examinations were performed on 1.5 T scanners with the patient in the supine position using a surface phased array coil. A 3-plane scout was obtained followed by half-Fourier single-shot fast spin-echo (SSFSE) T2-weighted images (TE = 60, slice thickness = 4–5 mm, 1 mm gap, matrix 192 × 256, flip angle 130–155, bandwidth 62 kHz, FOV 35–40 cm) in three orthogonal planes. Fat saturated SSFSE images in a single plane (with similar parameters) were obtained. Axial T1-weighted in-phase and opposed-phase gradient echo images (TR 205, TE 2.2 and 4.5, flip angle 80 degrees, slice thickness 5 mm, gap 2 mm, matrix 160 × 256, field of view 35 cm) were obtained in the axial plane.

### Data analysis

#### Clinical data at time of MRI

We recorded patient age, ovarian stimulation medication and dose, gestational age provided by the clinical record at the time of MRI, date of MRI examination, MRI indication, and prospectively interpreted MRI findings. Operative diagnosis, pathology diagnosis, and clinical follow up were recorded if available. The indications for MRI were right lower quadrant pain (*n* = 35), other localized sites of abdominal pain (*n* = 19), and abdominal pain site unspecified (*n* = 3), ovarian or adnexal cyst or mass (*n* = 3), and one case each of post-operative pain status post umbilical hernia repair, vaginal bleeding, fetal anomaly, and uterine sarcoma surveillance.

#### Imaging retrospective review

Two radiologists (gynecologic imager with 15 years experience and MRI specialist with 8 years experience) independently and blindly reviewed each study and recorded the size and the signal intensity of the ovaries. Size was recorded as greatest ovarian length in any image plane and diameter perpendicular to greatest length. Ovarian stromal signal intensity was recorded by comparing the two sides on non-contrast T1-weighted imaging (T1WI), T2-weighted imaging (T2WI) and fat saturated T2WI on a 5-point scale: right much brighter than left, right slightly brighter than left, ovaries symmetric in signal, left slightly brighter than right, left much brighter than right.

Other potentially important findings in the adnexa were recorded as: presence of multiple small peripheral follicles (yes or no), hemorrhagic corpus luteum or other hemorrhagic cysts (yes or no), multiple medium to large follicles as seen in stimulated ovaries (yes or no), hematosalpinx (yes or no), free fluid (yes or no), hemorrhagic free fluid (yes or no), and location of free fluid with respect to the ovaries. Dominant non-physiologic ovarian lesions, non-ovarian adnexal lesions, and non-gynecologic pathology were noted as free-text.

Each reviewer provided a final impression, including an assessment of the likelihood of ovarian/adnexal torsion. The reviewers knew that the patients were either stimulated or were pregnant, but were blinded to clinical indication for the MRI and outcome. The reviewers had participated in patient care at an interval of at least 2.5 years since the most recent MRI was performed.

For cases with discrepancy between the two reviewers regarding ovarian stromal signal intensity or final diagnosis (torsion versus non-torsion), a third reviewer recorded relative ovarian stromal signal intensity between the two sides and a final impression, without knowledge of the impression of the other two reviewers. If two of the three reviewers agreed, their concordant interpretation was used for analysis (*n* = 23). Although we had planned for a third round of review to allow for final diagnoses, this was not needed.

#### Outcome

Final diagnosis was determined using pathology if available (*n* = 9), surgical findings (*n* = 4), and if these were not available then MRI and clinical diagnosis from the emergency department visit or admission (*n* = 51). Clinical data was available through the time of discharge for 32 patients and at least through the time of delivery for 19 patients.

For the final analysis subgroups (*n* = 50), all final diagnoses of normal ovaries, asymmetric ovarian edema, and stimulated ovaries without torsion were made by MRI with chart review for further diagnosis (which did not change diagnosis in any of these instances). 5/6 diagnoses of torsion were based on histologic diagnosis and 1/6 was based on surgical findings (detorsed without oophorectomy/salpingectomy). PCOS diagnosis was based on clinical history/chart review.

#### Descriptive analysis

Due to small sample size, formal statistical analysis was not compared. Instead, detailed table gives individualized data. Analysis groups were based on ovarian final diagnosis: (1) normal ovaries, (2) asymmetric ovarian edema without torsion or stimulation, (3) stimulated ovaries without torsion, (4) ovarian torsion without stimulation, (5) ovarian torsion with stimulation, and (6) polycystic ovarian syndrome (PCOS). Because patients with PCOS can have enlarged ovaries without stimulation, those patients with clinical history of PCOS were considered separately. Another unexpected finding was that of ovarian edema without torsion or stimulation. These ovaries are also described in a separate group (post hoc decision).

Ovarian length and presence of signal asymmetry between right and left ovaries on T1WI, T2WI and fat-saturated T2WI were compared for each group. Differences in greatest ovarian length between torsed and non-torsed ovaries in the non-stimulated and stimulated populations are described. Absolute difference of greatest diameter, percent difference in greatest diameter ((larger ovary greatest diameter-smaller ovary greatest diameter)/larger ovary greatest diameter), and side to side difference in signal intensity were used as evaluation criteria. For one patient with final diagnosis of normal ovaries, only one ovary was visualized; the normal ovary was included in measurement analysis but side to side comparison was not performed.

Prospective MRI interpretation and retrospective reviewer diagnoses of torsion were compared with final diagnoses in cases of proven torsion and cases with suspected torsion based on MRI.

## Results

The 64 pregnant women included ranged in age from 18 to 41 years (mean 31 years, standard deviation 5 years). Gestational age range was 3–37 weeks (mean 19 weeks, standard deviation 9 weeks).

Initial reviewers’ interpretations were discrepant in 23/64 (36%) cases. Disagreements were signal intensity only (*n* = 14), impression only (*n* = 4), and signal intensity and impression (*n* = 5). For patients with torsion, discrepancies between the two initial reviewers occurred in 3/6 cases (50%), 1 involving concordant final impression but difference in interpretation of signal intensity, and 2 involving discrepancies in interpretation of both signal intensity and final impression. The consensus diagnosis was incorrect (stimulation without torsion) in 1/2 cases with discrepant final impressions. For one patient with stimulated ovaries without torsion, although prospective diagnosis was stimulated ovaries, retrospective diagnosis by both reviewers was normal ovaries (both ovaries measured at the upper limits of normal for size).

**Fig. 1:**
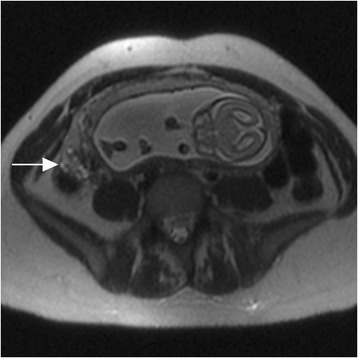
Normal ovary in pregnancy. Axial T2-weighted image with normal right ovary (arrow), demonstrating normal size, ovoid shape, stromal signal intensity, and small follicles

**Fig. 2:**
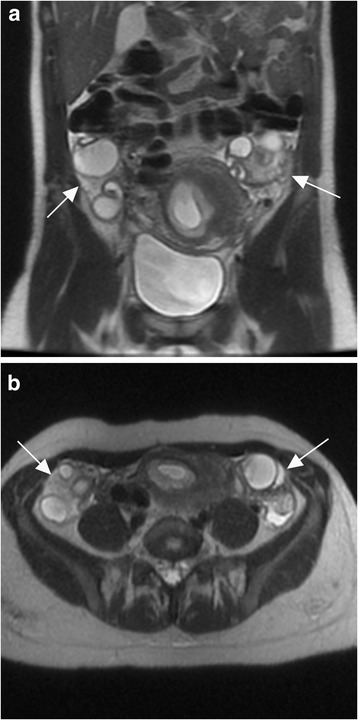
Stimulated ovaries without torsion. Coronal (a) and axial (b) T2-weighted MR images of a 37-year-old woman with right lower quadrant pain at 8 weeks gestational age demonstrate symmetrically enlarged ovaries (arrows) with increased T2 signal intensity of the ovarian stroma and multiple large follicles. The patient had a known diagnosis of ovarian hyperstimulation syndrome requiring paracenteses, was observed clinically, and was discharged without surgery

**Fig. 3:**
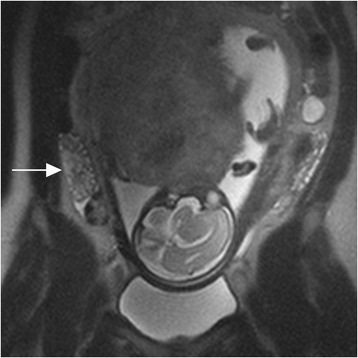
Ovarian torsion without stimulation. Coronal T2-weighted image in a 30-year-old woman with right lower quadrant pain at 28 weeks gestational age demonstrates an enlarged right ovary (arrow) with increased T2 stromal signal intensity and a normal appearing left ovary. The right ovary was torsed 720 degrees and was surgically removed

**Fig. 4:**
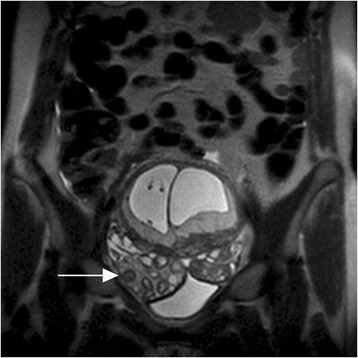
Ovarian torsion with stimulation. Coronal T2-weighted image in a 31-year-old woman with right lower quadrant pain at 11 weeks gestational age (dichorionic diamniotic twin gestation) demonstrates bilateral ovarian enlargement with asymmetric enlargement of the right ovary (arrow) and increased T2 signal intensity of the right ovarian stroma. Right ovarian/tubal torsion was surgically detorsed

The 50 women included in the size and signal intensity comparison had ovarian final diagnoses of normal ovaries (*N* = 27 women, Fig. 1), asymmetric ovarian edema without stimulation or torsion (*N* = 3), stimulated ovaries without torsion (*N* = 11, Fig. 2), ovarian torsion without stimulation (*N* = 3, Fig. 3), ovarian torsion with stimulation (*N* = 3, Fig. 4), and PCOS (*N* = 3). 5/6 diagnoses of torsion were confirmed histologically after oophorectomy and one was diagnosed surgically in a patient with stimulation who underwent surgical de-torsion without oophorectomy. Average greatest ovarian length for each group was 3.2, 4.5, 7.6, 5.6, 6, and 8.8 cm, respectively. 2/3 patients with PCOS had undergone ovarian stimulation.

**Fig. 5:**
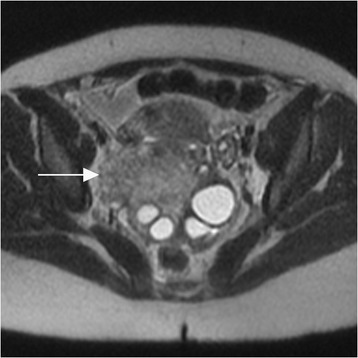
Stimulated ovary without torsion diagnosed as torsion by MRI. Axial T2 weighted MR image of the pelvis in a 31-year-old woman with abdominal pain and leukocytosis at 5 weeks 6 days gestational age demonstrates marked asymmetric enlargement of the right ovary (arrow) with increased right ovarian stromal signal intensity. Prospective and retrospective MRI diagnosis was ovarian torsion, but further history (not available at the time of image interpretation) revealed known asymmetric enlargement of the right ovary since the time of egg retrieval

Asymmetric signal on T1WI was seen in 0/27 (0%), 1/3 (33%), 2/11 (18%), 0/3 (0%), 1/3 (33%), and 1/3 (33%) patients, respectively. Asymmetric signal on T2WI was seen in 3/27 (12%), 3/3 (100%), 1/11 (9%), 2/3 (67%), 2/3 (67%), and 1/3 (33%) patients, respectively (Fig. 5). Signal on fat-saturated T2WI had similar findings (Table 1).

**Table 1:** Ovarian length and signal intensity in pregnant women undergoing MRI with respect to ovarian appearance, ovarian stimulation and torsion

Final diagnosis	Normal ovaries at time of retrospective review (*N* = 27 patients, *N* = 53 ovaries)	Not stimulated, not torsion but in retrospective review asymmetric ovarian edema (*N* = 3)	Stimulated without torsion (*N* = 11)	Torsion without stimulation (*N* = 3)	Torsion with stimulation (*N* = 3)	PCOS (*N* = 3) (2 stimulated, 1 non-stimulated)
Greatest ovarian diameter (cm)	3.2 +/− 0.6 (2.1–4.8)	4.5 +/− 0.6 (3.8–4.9)	7.6 +/− 2.8 (4.5–13.4)	5.6 +/− 0.9 (4.6–6.2)	6 +/− 1.5 (4.7–7.7)	8.8 +/− 4.8 (5.2–14.2)
Contralateral greatest ovarian diameter (cm)	2.5 +/− 0.6 (1.4–3.5)	3.1 +/− 0.3 (2.8–3.4)	5.4 +/− 1.2 (3.6–7.1)	3.7 +/− 1.1 (2.7–4.9)	3.9 +/− 1.7 (2.7–5.8)	6.1 +/− 2.4 (4.6–8.9)
Average difference in ovarian greatest diameter	0.6 +/− 0.5 (0–2.2)	1.3 +/− 0.8 (0.4–1.9)	2.2 +/− 2.4 (0.1–7.1)	2.6 +/− 1.3 (1.1–3.3)	2.1 +/− 0.3 (1.9–2.5)	2.7 +/− 2.4 (0.6–5.3)
Average % difference in ovarian greatest diameter	0.19 +/− 0.13 (0–0.51)	0.29 +/− 0.16 (0.11–0.4)	0.24 +/− 0.19 (0.02–0.63)	0.33 +/− 0.19 (0.21–0.55)	0.37 +/− 0.11 (0.25–0.44)	0.26 +/− 0.13 (0.12–0.37)
T1WI signal difference	0	1 (33%)	2 (18%)	0	1 (33%)	1 (33%) – T1 not available for 1 patient
T2WI signal difference	3 (12%)	3 (100%)	1 (9%)	2 (67%)	2 (67%)	1 (33%)
FS T2WI signal difference	3 (12%)	3 (100%)	1 (9%) – FS T2WI not available for one patient	2 (67%) – FS T2WI not available for one patient	2 (67%)	1 (33%) – FS T2WI not available for one patient

*Ovarian length data for individual examinations are given in the appendix table

The correct diagnosis of torsion was made prospectively in 5/6 (83%) cases and by our blinded reviewers in 3/6 (50%) cases. In patients without stimulation, torsion was diagnosed correctly in 3/3 (100%) cases prospectively and in 2/3 (67%) cases retrospectively. Asymmetric T2 signal was not seen in the 1 case for which torsion was not diagnosed retrospectively (possibly due to an ovarian fibroma diagnosed at pathology) but was seen for the 2 cases with correct retrospective diagnosis.

In patients with stimulation, torsion was diagnosed prospectively in 2/3 (67%) cases and by our reviewers in 1/3 (33%) cases. Asymmetric T2 signal was not seen for the 1 case that was not diagnosed prospectively (which was diagnosed as torsion by MRI 4 days later). However, asymmetric T2 signal was seen in the other two cases of torsion with stimulation, only one of which was diagnosed retrospectively (Table 2).

**Table 2:** Prospective and retrospective diagnoses for patients with torsion, torsion and stimulation, stimulation, asymmetric ovarian edema and PCOS

Indication for MRI	History of stimulation^a^	Prospective interpretation	Reviewer #1	Reviewer #2	Consensus reviewer	Asymmetric T2 signal	Final Diagnosis
LLQ pain	No	Left tubal torsion	Left tubal torsion	Left tubal torsion	N/A	Yes	Torsion
RLQ pain	No	Right ovarian torsion	Asymmetric enlargement of right ovary, possible torsion	Asymmetric enlargement of right ovary, possible PCOS	Right ovarian torsion	Yes	Torsion
RLQ pain	No	Possible right torsion	Edematous right tube, unclear etiology	Asymmetric ovaries, possible PCOS or torsion	N/A	No	Torsion (with possible ovarian fibroma at pathology)
LLQ pain	Yes	Stimulated ovaries	Stimulated ovaries	Stimulated ovaries	N/A	No	Torsion (diagnosed by MRI 4 days later) with necrosis at surgery/pathology
RLQ pain	Yes	Right ovarian torsion	Right ovarian torsion	Right ovarian torsion	Right ovarian torsion	Yes	Torsion
RLQ pain	Yes	Right torsion	Suggestive of right torsion	Stimulated ovaries	Stimulated ovaries	Yes	Torsion
RLQ pain	Yes	Asymmetric enlargement of right ovary	Right torsion vs. asymmetric stimulation	Right torsion	Stimulated ovaries	No	Stimulated ovaries
Abdominal pain	Yes	Right torsion	Right torsion	Right torsion	N/A	Yes	Asymmetric ovaries since time of egg retrieval
Pain (*N* = 6 patients)	Yes	Stimulated ovaries	Stimulated ovaries	Stimulated ovaries	N/A	No	Stimulated ovaries
Pain	Yes	Stimulated ovaries	Normal ovaries	Normal ovaries	N/A	No	Stimulated ovaries
Fetal anomaly	Yes	Stimulated ovaries	Stimulated ovaries	Stimulated ovaries	N/A	No	Stimulated ovaries
Uterine sarcoma surveillance	Yes	Resolving ovarian stimulation	Stimulation of PCOS	PCOS	N/A	No	Resolving ovarian stimulation
RLQ pain	No	Asymmetrically enlarged edematous right ovary	Normal versus early/intermittent right torsion	Normal	Asymmetric right ovarian edema	Yes	Asymmetric ovarian edema
RLQ pain	No	Asymmetrically enlarged edematous right ovary	Asymmetrically enlarged edematous right ovary	Right torsion	N/A	Yes	Asymmetric ovarian edema
RLQ pain	No	Right ovarian torsion	Right torsion	Right torsion	Right torsion	Yes	Edematous right ovary with a corpus luteum
RUQ pain	Yes	Stimulated ovaries	Stimulated ovaries	Stimulated ovaries	N/A	No	Stimulated ovaries, PCOS
RLQ pain	Yes	Stimulated ovaries, right pelvic hematoma status post egg retrieval	Stimulated ovaries, right pelvic hematoma	Stimulated ovaries	N/A	No	Stimulated ovaries, pelvic hematoma, PCOS
RLQ pain	No	Right torsion, PCOS	Possible right torsion	Edematous right ovary, PCOS	N/A	Yes	PCOS, normal ovaries seen 1 year later at tubal ligation

^a^history was not available to the retrospective blinded reviewers

*LLQ* left lower quadrant, *RLQ* right lower quadrant, *RUQ* right upper quadrant

**Fig. 6.**
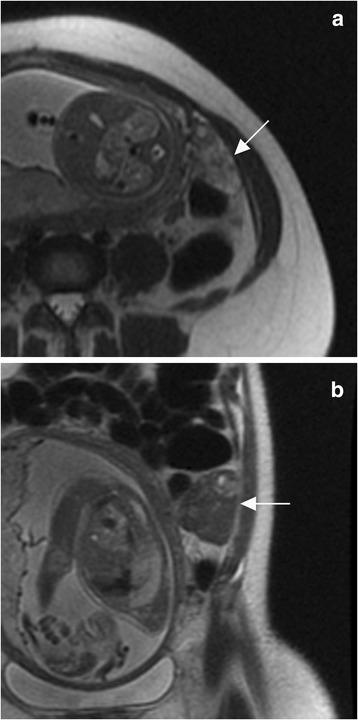
Stimulated ovary with torsion diagnosed as stimulated ovary without torsion by initial MRI. Initial axial T2 weighted image (a) in a 30-year-old female with left lower quadrant pain at 25 weeks gestational age demonstrates a mildly enlarged mildly T2 bright left ovary (arrow). Axial T2 weighted image 5 days later (b) demonstrates interval enlargement of the left ovary with dark T2 signal (and bright T1 signal, not shown), consistent with hemorrhagic necrosis. The patient underwent left oophorectomy.

In pregnant patients with stimulation without torsion, prospective interpretation was incorrectly torsion in 1/11 (9%) cases and by our reviewers in 2/11 (18%) cases (Table 2, Fig. 6). The case which was incorrectly diagnosed as torsion both prospectively and retrospectively had asymmetric T2 signal, and the case that was diagnosed incorrectly only retrospectively did not have asymmetric T2 signal (Table 2).

Three women had asymmetric ovarian edema without torsion or stimulation. The greatest ovarian lengths for these patients ranged from 3.8–4.9 cm. Two of these patients were followed clinically with resolution of symptoms. For the third patient, both prospective and retrospective diagnoses were ovarian torsion. The patient was taken to surgery and found to have an edematous ovary with a corpus luteum.

Of the three women with PCOS, two had undergone ovarian stimulation and had greatest ovarian lengths of 14 cm (6 weeks pregnant) and 7 cm (recently underwent embryo transfer). The patient with PCOS who had not undergone stimulation had a final diagnosis of asymmetric ovarian edema by MRI at 29 weeks gestation, was admitted briefly for pain control, delivered at term, and was noted to have a normal ovary at the time of surgical tubal ligation 1 year after the MRI.

8/27 (30%) patients with normal ovaries had other acute non-ovarian/adnexal findings on MRI, including appendicitis, obstructing ureteral stone, degenerating fibroid, and membranes bulging through the cervix. The 14/64 patients excluded for other ovarian/adnexal lesions included dermoid, ovarian cyst, ruptured hemorrhagic cyst or corpus luteum, cystadenofibroma, and degenerating fibroid obstructing ovarian venous outflow. In total, 13/64 (20%) patients had acute non-torsion gynecologic or non-gynecologic findings (Table 3).

**Table 3:** Other diagnoses

Diagnosis	Number of patients
Appendicitis^b^	2^a^
Degenerating fibroid^b^	5
Dermoid	6^a^
Membranes bulging through cervix at 20 weeks gestation	1
Cystadenofibroma	2
Ruptured hemorrhagic cyst or corpus luteum	3^a^
Obstructing ureteral stone^b^	2
Ovarian cyst	3
Total	22 patients with 24 diagnoses

^a^1 patient had both appendicitis and dermoid and one patient had both ruptured corpus luteum and dermoid

^b^Patients with appendicitis, degenerating fibroid, and obstructing ureterolithiasis with normal ovaries on MRI were included in the analysis of normal ovaries (*N* = 8)

## Discussion

When ultrasound is non-diagnostic for pregnant women with pelvic pain, MRI is frequently performed. In pregnancy, gadolinium is typically not utilized, thus findings other than asymmetric enhancement on MRI need to be utilized for diagnosis of torsion. Patients with ovarian stimulation complicated by torsion can be difficult to diagnose since the ovaries are typically enlarged in this setting. Although all torsed ovaries were larger on the affected side, as expected, absolute size was not a useful criterion in distinguishing stimulated ovaries with and without torsion, since asymmetric size was commonly present in stimulated ovaries.

We hypothesized that asymmetric ovarian edema would be a useful MRI finding for these patients. In our population, pregnant patients with torsion both without and with stimulated ovaries were more likely to have asymmetric ovarian edema than pregnant stimulated patients without torsion (67% versus 9%). However, for stimulated patients, asymmetric ovarian edema was not adequate to confidently diagnose or exclude torsion. Asymmetric ovarian edema was also seen in non-torsed ovaries without stimulation, one of which was thought to be torsion both prospectively and retrospectively.

In the setting of inconclusive ultrasound findings, the diagnosis of ovarian torsion is frequently made by a combination of imaging findings and clinical presentation. Pain out of proportion to imaging findings on ultrasound suggests a diagnosis of torsion. Therefore, we expected that our reviewers, blinded to clinical presentation, would not perform as well as those who made prospective interpretations with access to clinical presentation and prior imaging at the time of interpretation. Thus it is not surprising that the diagnosis of ovarian torsion in patients with stimulation was made prospectively in 2/3 cases and retrospectively (blinded to clinical presentation and prior imaging) in only 1/3 cases. The diagnosis of torsion was inaccurately attributed to stimulated ovaries without torsion in 1 case prospectively and in 2 cases retrospectively, further demonstrating the challenge of diagnosing torsion by MRI in this population.

The diagnosis of torsion was incorrectly attributed to possible PCOS in two non-stimulated patients retrospectively. One patient with resolving stimulation without torsion was also diagnosed retrospectively incorrectly as PCOS. And one patient with PCOS was incorrectly retrospectively diagnosed as torsion due to asymmetric ovarian edema. Because patients with PCOS also have enlarged ovaries at baseline, torsion can be difficult to diagnose in this population, particularly in patients with PCOS who undergo ovarian stimulation. The two stimulated patients with PCOS in our series had wide ranging greatest ovarian sizes of 14 cm and 7 cm. Ovarian stimulation in patients with PCOS has been reported to increase the risk for ovarian hyperstimulation syndrome [15]. However, a larger review of the MRI appearance of ovaries in patients with PCOS undergoing ovarian stimulation is needed to evaluate the effect of PCOS on ovarian size and signal intensity in this setting.

The limitations of this study include the retrospective nature of the study, small sample size, variable follow up data, and potential for recall bias. In some cases with clinical follow-up rather than surgery, a chronic torsion could be missed. These data comprise 12 years of pelvic MRI in women of reproductive age at an urban academic medical center with a busy obstetric and gynecologic service. In this setting, where ultrasound is typically performed as the initial imaging study and MRI is pursued only in those cases where ultrasound is non-diagnostic, we found only 6 cases of ovarian torsion in pregnant women imaged with MRI. It was beyond the scope of this study to assess all patients with ovarian torsion during the time period, thus our population is subject to inclusion bias, and does not represent the full spectrum of appearance of torsion, since most of these patients are diagnosed with a combination of clinical and ultrasound findings.

One fifth of patients in our study had acute non-torsion findings diagnosed by MRI, such as degenerating fibroid, appendicitis and obstructing ureterolithiasis. At our institution, most patients undergoing MRI in pregnancy undergo ultrasound first and only pursue MRI if the ultrasound is non-contributory to diagnosing the presenting symptoms. Therefore, MRI provided additional clinically important diagnostic information.

## Conclusions

Symmetrically or asymmetrically enlarged and edematous ovaries can be seen in the setting of ovarian stimulation. However, without knowledge of clinical presentation, torsed and non-torsed stimulated ovaries could not be distinguished with these findings. In our small sample, non-contrast enhanced MRI (typically performed after inconclusive ultrasound) could not be used to confidently diagnose or exclude ovarian torsion in pregnant patients with stimulated ovaries. However, MRI is useful for diagnosing and excluding other causes of acute pain and pelvic pathology in this patient population.

## Appendix

Table 4.

**Table 4:** Ovarian length data for stimulated, torsed, stimulated torsed, asymmetrically edematous, and polycystic ovaries

	Larger ovarian diameter (cm)	Smaller ovarian diameter (cm)	Difference in right and left ovarian diameter	Percent difference in right and left ovarian diameter
Stimulated non-torsed	5.2	5.1	0.1	0.02
	13.4	6.3	7.1	0.53
	10	3.7	6.3	0.63
	5.2	5.1	0.1	0.02
	10.6	7.1	3.5	0.33
	7.4	5.8	1.6	0.22
	8.1	6.3	1.8	0.22
	5.9	5	0.9	0.15
	5.8	4.5	1.3	0.22
	7.4	6.7	0.7	0.09
	4.5	3.6	0.9	0.2
Torsed non-stimulated	6	2.7	3.3	0.55
	4.6	3.5	1.1	0.24
	6.2	4.9	1.3	0.21
Torsed stimulated	5.7	3.2	2.5	0.44
	4.7	2.7	2	0.43
	7.7	5.8	1.9	0.25
Asymmetrically edematous without torsion or stimulation	3.8	3.4	0.4	0.11
	4.7	2.8	1.9	0.4
	4.9	3.2	1.7	0.35
PCOS, stimulated	14.2	8.9	5.3	0.37
	7	4.9	2.1	0.3
PCOS, non-stimulated	5.2	4.6	0.6	0.12

## Abbreviations

ADC: Apparent diffusion coefficient
MRI: Magnetic resonance imaging
PCOS: Polycystic ovary syndrome
SSFSE: Single-shot fast spin-echo
T1WI: T1-weighted imaging
T2WI: T2-weighted imaging
